# Primary Hyperparathyroidism and Cardiovascular Disease: An Association Study Using Clinical Natural Language Processing Systems and Big Data Analytics

**DOI:** 10.3390/jcm12216718

**Published:** 2023-10-24

**Authors:** Pedro Iglesias, Javier Arias, Guillermo López, Iago Romero, Juan J. Díez

**Affiliations:** 1 Department of Endocrinology and Nutrition, Hospital Universitario Puerta de Hierro Majadahonda, Instituto de Investigación Sanitaria Puerta de Hierro Segovia de AranaMajadahonda, 28222 Madrid, Spain; juanjose.diez@salud.madrid.org; 2 Department of Medicine, Universidad Autónoma de Madrid, 28049 Madrid, Spain; 3 MedSavana S.L., 28004 Madrid, Spain; jarias@savanamed.com (J.A.); glopez@savanamed.com (G.L.); iromero@savanamed.com (I.R.)

**Keywords:** primary hyperparathyroidism, hypertension, dyslipidemia, diabetes mellitus, smoking habit, stroke, atrial fibrillation, ischemic heart disease

## Abstract

Primary hyperparathyroidism (PHPT) seems to be associated with different cardiovascular diseases (CVDs). We evaluated the association of PHPT with major CV risk factors (CVRFs) and CVDs by using artificial intelligence (AI) tools. An observational and retrospective study was conducted using data from the electronic health records (EHRs) of the Hospital Universitario Puerta de Hierro Majadahonda (Spain). Of a total of 699,157 patients over 18 years of age studied (54.7% females), 6515 patients (0.9%; 65.4% women; mean age 67.6 ± 15.9 years) had a diagnosis of PHPT. The overall frequencies of hypertension, dyslipidemia, diabetes mellitus, and smoking habit in the cohort of patients with PTHP were all significantly (*p* < 0.001) higher than those found in patients without a diagnosis of PTHP. The total frequency of stroke, ischemic heart disease, atrial fibrillation, deep vein thrombosis, and pulmonary embolism in the cohort of PHPT patients were significantly (*p* < 0.001) higher than that found in patients without the diagnosis of PHPT. A multivariate regression analysis showed that PHPT was significantly (*p* < 0.001) and independently associated with all the CVDs evaluated. Our data show that there is a significant association between the diagnosis of PHPT and the main CVRFs and CVDs in our hospital population.

## 1. Introduction

Primary hyperparathyroidism (PHPT) is a disease characterized by the autonomous production of parathyroid hormone (PTH) by the parathyroid glands. The excess PTH associated with PHPT seems to affect the cardiovascular (CV) system, although the exact relationship between this condition and CV disease (CVD) is not fully understood. PHPT might contribute to the development of CVD through several mechanisms. One of the most important factors would be the effect of excess PTH on calcium metabolism. Hypercalcemia can promote arterial calcification, increasing the risk of atherosclerosis and coronary heart disease. In addition, hypercalcemia might alter endothelial cell function, which is a predisposing factor for the development of CVD [1]. Other mechanisms that have been considered are the direct effect of PTH on the proliferation of vascular and cardiac musculature [2], the action of intracellular calcium mediated by PTH on insulin sensitivity [3], and dysfunction of the renin–angiotensin–aldosterone system [4,5].

Different observational studies suggest that PHPT may be associated with different CVD, such as hypertension, arrhythmias, vascular and valvular calcification, increased carotid intima-media thickness, increased carotid and aortic vascular stiffness, left ventricular hypertrophy, and diastolic dysfunction [6,7,8]. However, at this time, there are no clinical data to support routine CV evaluation in patients diagnosed with PHPT.

Some studies have shown conflicting results regarding the effect of parathyroidectomy on the reduction of insulin resistance and related complications in patients with PHPT [9,10]. On the other hand, the effect of parathyroidectomy in patients with PHPT on the development of CVDs and CV mortality is not completely defined [11,12,13].

In recent years there has been a growing interest in using artificial intelligence (AI) tools that can extract valuable information from the large amount of data generated in healthcare centers [14,15,16,17,18]. Most of the clinical information in electronic health records (EHRs) is in free text and, due to the large number of data, is poorly accessible by traditional manual review. This manual processing of information is time-consuming, costly, and error prone, and it imposes inherent limitations on the volume and type of information that can be extracted [19]. Natural language processing (NLP) can be utilized to automatically derive value from the clinical notes. NLP is a field that combines linguistics and artificial intelligence to allow computers to comprehend and interpret text, and it is being widely applied to extract and gain insight into hidden information in clinical notes [20]. Machine learning enhanced NLP has enabled information extraction (IE) from unstructured free-text documents in a wide range of clinical data sources such as clinic letters, progress notes, discharge summaries, and test reports. This technology can improve care quality in multiple departments [21].

In this context, recent advances in clinical natural language processing (cNLP), big data, and AI programs make it possible to enable the quick and easy extraction of clinical data regarding many patients with real, verifiable, and direct information from clinical practice. The cNLP applications in pathology are designed to assist pathologists in clinical decision making, processing patient data, and analyzing medical knowledge [22]. They are not meant to replace pathologists but, rather, enhance their expertise. In the future, the focus should be on sharing data and linguistic resources to advance NLP in pathology [23]. Additionally, machine learning algorithms are being used to create innovative diagnostic tools that rapidly analyze images from tissue samples, especially in the emerging field of digital pathology, where combining digital images with textual reporting could be a promising direction for the future [24].

To date, few studies have specifically evaluated the association of PHPT with both CV risk factors (CVRFs) and CVDs and, to the best of our knowledge, data extraction was performed in traditional ways. Therefore, this study does not only focus on an underrepresented topic, but also aims at increasing the available data by extracting clinically relevant information from EHR free text using modern methods. Therefore, we used cNLP and big data tools applied to the database from the Hospital Universitario Puerta de Hierro Majadahonda (HUPHM) with the aim of analyzing the frequencies of different CVRFs and CVDs in patients diagnosed with PHPT by using cNLP and big data tools. A secondary objective was to analyze whether the associations found between PHPT and CVDs were independent of the classic CVRFs.

## 2. Material and Methods

### 2.1. Type of Research Study

An observational, retrospective, non-interventional study was conducted in adult patients using data from the EHRs of HUPHM. The study comprised clinical data collected between 25 September 2008 and 22 March 2023. These data came from documents generated in hospitalization, emergency, and outpatient departments. The present study was approved by the Clinical Research Ethics Committee of the HUPHM (code PI 169/23).

### 2.2. Study Definitions and Outcome

The variables processed by AI were the following: age, sex, PHPT, hypertension, dyslipidemia, diabetes mellitus, smoking, stroke, ischemic heart disease, atrial fibrillation, deep vein thrombosis, and pulmonary embolism.

The study examined the occurrence of CVRFs (hypertension, dyslipidemia, diabetes mellitus, and smoking) as well as CVDs (stroke, ischemic heart disease, atrial fibrillation, deep vein thrombosis, and pulmonary embolism) in individuals with a diagnosis of PHPT and those without this medical condition.

These variables were assessed with respect to the age and gender of the patients. Patients were not classified as hypertensive, diabetic, or dyslipidemic based on clinical criteria. Instead, these diagnoses were considered valid if they were documented in the patient’s EHR. Therefore, no specific definition was taken into account for each of the terms evaluated, but was based on their presence in the EHR and, consequently, on the criteria of the responsible physician.

The primary outcome of our observational study was to know the prevalence of CVRFs and CVDs in patients with and without PHPT.

### 2.3. Data Extraction and Clinical NLP Processing

The data relevant to the project were extracted by the HUPHM information technology department in collaboration with Savana, a medical company that provides clinical natural language processing (cNLP) services to create real-world evidence [25,26]. Before the data could leave the HUPHM to Savana’s servers via a secure file transfer protocol, an important procedure needed to be executed beforehand during which personal health information was protected by a pseudonymization step facilitated by Savana’s data integration software version 4.0. Once the data were adapted to the respective data model, a baseline processing of Savana’s patented clinical NLP pipeline, EHRead^®^ technology, was executed to detect clinically relevant information in the free text section of the provided EHRs. EHRead^®^ Technology is a complex cNLP pipeline combining a rich set of NLP techniques in a big data processing pipeline and has been successfully applied in a wide range of real-world evidence studies [14,15,16,17,18,27]. The outcome of the baseline processing is a structured database in which clinically relevant information extracted from the EHRs’ free text was added to the information coming from structured data sources.

For the situation that the baseline processing does not contain the information required to answer a question for a specific research study, Savana developed Savana Manager version 4.0 (SMv4), a piece of software that enables the clinician to design a strategy to enrich existing data with additional information detected in the free text of the study population’s EHRs. The idea behind SMv4 is to offer clinicians a simplified fast-track option to customize a cNLP pipeline to their study needs for the extraction of clinically relevant information from free text to respond to their study questions. SMv4 contains a subset of functionalities of EHRead^®^ technology mainly consisting of a customizable layer of named-entity recognition including named-entity disambiguation plus baseline machine learning models covering, for example, negation and temporality detection. Using a user-friendly user interface, SMv4 guides the clinician to specify the list of clinical entities that should be detected in the free text. The underlying terminology is based on SNOMED CT (https://www.snomed.org/ (accessed on 29 January 2023), enriched with clinical concepts defined by Savana. Once the configuration is submitted, Savana executes the cNLP process resulting in the delivery of a database containing information from structured data sources combined with the additional information from the EHRs’ free text regarding the clinical variables and their attributes asked for by the clinician. This database is then uploaded to SMv4 where the clinician can analyze and visualize the enriched data. If needed, the data can be exported to various convenient formats using different options of data aggregation (e.g., level of patient, level of record, and age range) for downstream analyses.

For the scope of this study, the variables specified in the section ‘Study variables’ were detected in the free text using a named-entity recognition approach. As additional layers, negation and temporality detection were applied. The machine learning model to detect negation is a combination of a rule-based layer with a binary convolutional neural network [28] trained on real Spanish EHRs and evaluated against a rich set of reference standards. This model classifies each clinical entity as being affirmative or non-affirmative based on its lexical and semantic context. The temporality detection is conducted by a NLP module that consists of various layers that work in combination to assign dates to clinical entities. The first layer is a named-entity detection engine responsible for the detection of any mentioning of dates in the free text of EHRs. Subsequently, a relationship model based on a Bi-LSTM [29] decides if a detected date is related to a detected clinical entity or not. In addition, a normalization layer takes care of converting different date formats as written in the EHRs’ free text into a normalized representation. The last step of the NLP processing, the post-processing step, conducts several quality control operations and combines the output from the different NLP modules into a final database. After the cNLP processing, HUPHM authors validated the results of the tool and the performance of the technology.

The aim of this assessment was to validate the accuracy of the EHRead^®^ technology in identifying records that contained references to “primary hyperparathyroidism” and its associated variables. A total of 119 documents were subject to manual verification, establishing a reliable manual annotation and review process that served as the gold standard. Savana’s performance was assessed using the gold standard created by experts as the evaluation resource. In other words, the accuracy of Savana in identifying records that mentioned the disease under study and its related variables was measured in comparison to the gold standard. This performance was determined using standard metrics, including precision (P), recall (R), and the F-score, which represents the harmonic mean of these two metrics [30].

Precision, which reflects the accuracy of the information retrieved by the system, was calculated using the formula P = tp/(tp + fp). Recall, an indicator of the amount of information retrieved by the system, was calculated as R = tp/(tp + fn). The F-score was determined as F = 2 × precision × recall/(precision + recall), and it served as a comprehensive measure of the system’s overall information retrieval performance. In all instances, true positives (tp) represented correctly identified records, false negatives (fn) represented records that were not identified, and false positives (fp) represented records that were incorrectly retrieved.

The linguistic evaluation of the variable “primary hyperparathyroidism” conducted in the context of this study yielded precision, recall, and an F-score, all equal to 1. These scores indicate that the identification of primary hyperparathyroidism (PHPT) diagnoses in the study population was highly accurate. It is worth noting that, in this study, the variable PHPT encompassed all patients with this diagnosis, regardless of their parathyroid hormone (PTH) levels, the degree of hypercalcemia (whether hyper- or normocalcemic), or whether the patient had received treatment with calcimimetics and/or surgery.

The other variables examined demonstrated F-scores exceeding 0.75. For CVRFs, the F-score values were 1 for hypertension, 0.93 for dyslipidemia, 0.75 for diabetes mellitus, and 0.92 for smoking. Regarding CVDs, these values were 0.81 for stroke, 0.91 for ischemic heart disease, 0.97 for atrial fibrillation (AF), 0.80 for deep vein thrombosis, and 0.90 for pulmonary embolism. These F-score values provide insights into the accuracy of identifying these variables within the study.

### 2.4. Statistical Analysis

A descriptive analysis was conducted for all the evaluated variables. Qualitative variables were presented in terms of their absolute frequencies and percentages. To assess the association and compare proportions between qualitative variables, the chi-square test was employed. The relative risk of CVRF and CVD in patients diagnosed with PHPT in comparison to subjects without PHPT was estimated using the odds ratio (OR). Furthermore, a multivariate logistic regression analysis was performed to evaluate the OR for various CVDs in patients with PHPT, while considering potential confounding factors such as age, gender, and classical risk factors like smoking, diabetes, dyslipidemia, and hypertension. In all instances, differences for which the associated *p*-value from the contrast test was less than 0.05 were regarded as statistically significant. This rigorous statistical approach allowed for a comprehensive analysis of the variables and their associations within the study.

## 3. Results

### 3.1. Study Patients

A total of 699,157 patients aged 18 years and older were registered in the Savana Manager v4.0 tool, of whom 382,597 (54.7%) were females and 316,560 (45.5%) were males. Of the total population studied, 6515 patients (0.9%) had a diagnosis of PHPT. The mean age at the diagnosis of PHPT was 67.6 ± 15.9 years. The prevalence of PHPT was significantly higher in women than in men (65.4% vs. 34.6%; *p* < 0.001). Lastly, women were significantly older than men at diagnosis of PHPT (68.2 ± 16.0 vs. 66.5 ± 15.5 years; *p* < 0.001).

### 3.2. Frequency of Cardiovascular Risk Factors

The total frequency of CVRFs in the cohort of patients studied with and without PHPT is summarized in Table 1.

The overall frequencies of hypertension, dyslipidemia, diabetes, and smoking habit in the cohort of patients with PHPT were 73.7%, 49.0%, 31.3%, and 28.3%, respectively, which were all significantly (*p* < 0.001) higher than those found in patients without a diagnosis of PHPT (31.7%, 18.4%, 9.3%, and 22.7%, respectively). These higher frequencies were also observed for all CVRFs in both women and men separately (*p* < 0.001) (Table 1).

### 3.3. Frequency of Cardiovascular Diseases

The total frequency of CVDs in the cohort of patients studied with and without PHPT is summarized in Table 2.

The total frequencies of stroke, ischemic heart disease, atrial fibrillation, deep vein thrombosis, and pulmonary embolism in the cohort of PHPT patients studied were 12.2%, 15.3%, 23.0%, 14.6%, and 8.4%, respectively, which were significantly (*p* < 0.001) higher than those found in patients without the diagnosis of PHPT (3.7%, 5.6%, 5.7%, 4.2%, and 2.5%, respectively). These higher frequencies for all CVDs were observed equally in women and men (*p* < 0.001) (Table 2).

### 3.4. Multivariate Regression Analysis

**Figure 1:**
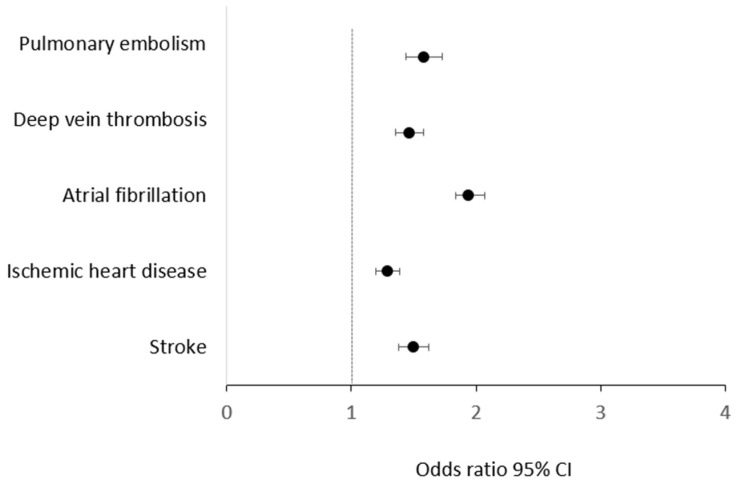
Forest plot showing odds ratios and 95% confidence intervals (CI) for the association of primary hyperparathyroidism (PHPT) and different CVDs in multivariate regression analysis adjusted for age, gender, hypertension, dyslipidemia, diabetes, and smoking habit.

Univariate regression analysis showed that PHPT was significantly associated with all CVDs analyzed. Multivariate regression analysis, adjusted for age, sex, and major CVRFs such as hypertension, dyslipidemia, diabetes, and smoking habit, showed a significant (*p* < 0.001) and independent association between PHPT and the CVDs analyzed separately (Table 3; Figure 1).

## 4. Discussion

The results of the present study reveal a significant association between the diagnosis of PHPT and major CVRFs as well as CVDs within the patient population of our hospital. Moreover, even after adjusting for variables associated with CV morbidity, such as age, gender, and major CVRFs, the diagnosis of PHPT remained significantly and independently associated with the main CVDs.

The results of our study showed a similar prevalence of PHPT (0.9%) to that previously reported in different surveys (0.2–1.3%), with a greater predilection for the female gender and, generally, detected in the seventh decade of life [31].

Different studies have reported an association between PHPT and some CVRFs. Patients with PHPT may be at increased risk for the development of hypertension, but causality has not been clearly established. PHPT independently predicted the risk of hypertension in a population of over 37,000 inpatients from a large national database in the US [32]. In this study, a diagnosis of PHPT was associated with a 30% increased risk of hypertension showing a prevalence of hypertension of 69% in PHPT patients vs. 39% reported in patients without PHPT (*p* < 0.0001). These data were similar to those found in our study (73.7% vs. 31.7%; *p* < 0.001), supporting such association. Proposed pathogenic mechanisms in this association include activation of the renin–angiotensin system and alterations in vascular structure and function [33]. Other factors, such as genetic predisposition, advanced age, and chronic kidney disease associated with PHPT hyperparathyroidism, may also contribute to hypertension in these patients. The results of some studies support an improvement in blood pressure and/or the presence of hypertension after parathyroidectomy, while others have shown no beneficial effect on blood pressure after parathyroid surgery [13,33,34].

Although an association between PHPT and dyslipidemia has been reported [35,36], the exact relationship between the two entities is not fully understood. The findings of our study confirm these data showing that dyslipidemia is 2.7 times (*p* < 0.001) more common in patients with PHPT than in patients without this diagnosis. PHPT patients show higher levels of total cholesterol, LDL (low-density lipoprotein) cholesterol, and triglycerides, and lower levels of HDL (high-density lipoprotein) cholesterol compared with individuals without this condition. Likewise, PHPT has been associated with insulin resistance, which, in turn, can alter lipid metabolism, increasing triglyceride levels and decreasing HDL cholesterol levels [9]. PHPT may be also associated with a chronic low-grade inflammatory state that negatively affects lipid metabolism and promotes the formation of smaller and denser LDL particles [37]. Lastly, excess of PTH may increase oxidative stress, alter lipoprotein levels, and promote oxidation of LDL particles, rendering them more atherogenic [38].

There is some evidence of an association between PHPT and type 2 diabetes mellitus (T2DM), but the exact nature of their relationship is not fully understood and more research is needed to better understand the underlying mechanisms. Some studies have found that people with PHPT may have an increased risk of developing insulin resistance and, therefore, an increased risk of developing T2DM [39]. However, other studies in patients with normocalcemic PHPT have failed to demonstrate an association with insulin resistance [40]. In our study, the prevalence of T2DM was 3.4-fold (*p* < 0.001) higher in PHPT patients than in non-PHPT patients, supporting this association. Elevated PTH levels can adversely affect glucose metabolism and insulin homeostasis. Both intracellular hypercalcemia and hypophosphatemia are likely to affect insulin receptor expression and response. One proposed pathogenic mechanism is that an increase in PTH levels is accompanied by an increase in intracellular calcium, thereby reducing insulin-stimulated tissue glucose uptake, leading to insulin resistance, followed by glucose intolerance and, ultimately, diabetes [41]. It remains unclear why some patients with PHPT improve their insulin sensitivity after parathyroidectomy while others do not [39,42].

Smoking has been reported to be associated with lower plasma PTH levels and decreased bone mineral density (BMD) in healthy subjects. Similarly, in PHPT, smoking is associated with lower plasma PTH and higher plasma phosphate levels [43]. To the best of our knowledge to date, no direct relationship has been established between primary hyperparathyroidism and smoking. Our study showed a significantly higher prevalence of smoking in patients with PHPT than in patients without PHPT. The clinical significance of this finding deserves confirmation by other studies, which should also consider the possibility of a causal relationship between both processes. However, the fact that this is a sub-group of hospital patients with an increased risk of CVD means there may be better documentation of smoking habits than in other groups.

PHPT may be related to CVD, increasing the risk of coronary heart disease and other CV events, through hypercalcemia that favors endothelial dysfunction, thrombosis, calcification of the myocardium, heart valves and coronary arteries, and the development of hypertension, left ventricular hypertrophy, and arrhythmias [44,45,46]. On the other hand, PTH has positive inotropic and chronotropic effects and favors the development of left ventricular hypertrophy [46,47]. Furthermore, as we have shown in our study, PTH is associated with other CVRFs such as hypertension, dyslipidemia, diabetes, and smoking.

A recent analysis of comorbidities and clinical outcomes performed in 16,374 Swedish adults patients with PHPT showed that the risk of CV events was significantly increased by 45% in patients with PHPT compared with a matched control group [44]. As in the Swedish population, our study performed in the Spanish population also showed an association between PTH and CVDs. We herein report that in our hospital population, the diagnosis of PTH was significantly and independently associated with stroke, ischemic heart disease, atrial fibrillation, deep vein thrombosis, and pulmonary embolism. This is important because, as recently reported, parathyroidectomy appears to be associated with clinical benefit in terms of reduced long-term CV events, even in elderly patients, compared with non-operative management. This finding is relevant for surgical decision making, especially in patients with a long life expectancy [44,48].

The use of AI in medical diagnostic and therapeutic procedures, as well as the use of AI to review computerized medical data, is an extremely attractive topic in healthcare [49]. In light of upcoming technological advances and research directions, the use of AI may be of critical importance and great interest for future research. The results of our study, using AI techniques, confirm the association of PHPT with CVDs. These findings oblige us to consider PHPT as another CVRF and to adopt measures aimed at controlling and/or curing PHPT, especially in those patients with a higher CV risk. AI could also help to determine whether there is an association between asymptomatic normocalcemic PHPT and CVDs. At present, there is controversy regarding the data supporting the reversibility or improvement of CV alterations after parathyroidectomy. The use of AI will help us to know whether patients with PHPT show a reduction in CV risk after cure with parathyroidectomy and to detect those patients who may benefit from it. It will also help us to know whether medical treatment with calcimimetics (cinacalcet) could have a beneficial effect on the CV risk associated with PHPT, especially in patients with hypercalcemia in whom parathyroidectomy is not indicated due to associated comorbidity.

The main strength of our study lies in the extraction of real-world data and the achievement of a substantial and unbiased sample size. By implementing big data techniques and utilizing Savana’s EHRead technology, we were able to assess a large volume of information, effectively read, process, and organize the unstructured text in EHRs, and, ultimately, convert it into structured data. The dataset examined included diagnoses from nearly 700,000 patients throughout the study, ensuring the reliability and impartiality of the findings derived from real-world clinical practice.

A limitation of our study is the inability to analyze differences according to the etiology, duration, and severity of PHPT. Lack of laboratory data is another limitation of the study. Although the database included the date of the first document in which the variable appeared, we cannot assume with complete certainty that this date corresponds to the exact date of diagnosis of both PHPT and CVDs. Consequently, we were unable to establish a definitive temporal relationship between the period of exposure to elevated PTH levels and the diagnosis of the CVD analyzed. Our study did not allow as to assess differences according to serum levels of calcium or phosphorus, the use of drugs such as calcimimetics, bisphosphonates, calcifediol, thiazide diuretics, or the changes induced by parathyroidectomy. Another limitation to consider is that Savana exclusively extracts information from electronic health records (EHRs) without making generalized inferences. As a result, the system may not have the capability to identify subtle patterns or draw conclusions that are not explicitly documented in the EHRs. In addition, the accuracy of the results obtained by the system depends on the correct diagnoses recorded in the patient’s medical record. Finally, the study cohort was a sample of patients from our own hospital and may not be representative of the general population. In this sense, our study, by focusing on a specific patient population within a hospital setting, rather than a community-based sample, could explain the high prevalence of smoking among PHPT patients. It should be kept in mind that a higher prevalence of multiple health conditions, particularly the association with CVD, could contribute to a higher number of patients seeking or being referred to the hospital for additional medical care.

## 5. Conclusions

In conclusion, the current study, leveraging NLP and big data analytics, demonstrates that PHPT is closely linked with significant CVRFs including hypertension, dyslipidemia, diabetes, and smoking habit. Furthermore, PHPT emerges as an independent CVRF, and its connection with other CVRFs substantially amplifies the CV risk in these patients. These findings underscore the importance of recognizing and addressing PHPT as a critical factor in CV health. Furthermore, the concordance of our results with the majority of previously published studies provides valuable insights into the potential application of AI methods in real-world data and information analysis.

## Figures and Tables

**Table 1:** Prevalence of cardiovascular risk factors analyzed in the cohort of patients studied with and without primary hyperparathyroidism (PHPT) (chi-square test).

		PHPT (*n* = 6515)	Non-PHPT (*n* = 692,642)	Total (*n* = 699,157)
Hypertension	women	2969 (45.6%) *	115,098 (16.6%)	118,067 (16.9%)
	men	1834 (28.1%) *	104,217 (15.1%)	106,050 (15.2%)
	total	4803 (73.7%) *	219,315 (31.7%)	224,118 (32.1%)
Dyslipidemia	women	2025 (31.1%) *	70,214 (10.1%)	72,239 (10.3%)
	men	1169 (17.9%) *	57,102 (8.3%)	58,271 (8.4%)
	total	3194 (49.0%) *	127,316 (18.4%)	130,510 (18.7%)
Diabetes mellitus	women	1192 (18.3%)	32,069 (4.6%)	33,261 (4.8%)
	men	848 (13.0%) *	32,650 (4.7%)	33,498 (4.7%)
	total	2040 (31.3%) *	64,719 (9.3%)	66,759 (9.5%)
Smoking habit	women	872 (13.4%) *	66,757 (9.6%)	67,629 (9.7%)
	men	972 (14.9%) *	90,328 (13.1%)	91,300 (13.0%)
	total	1844 (28.3%) *	157,085 (22.7%)	158,929 (22.7%)

* *p* < 0.001 (PHPT vs. non-PHPT).

**Table 2:** Prevalence of cardiovascular diseases analyzed in the cohort of patients studied with and without primary hyperparathyroidism (PHPT) (chi-square test).

		PHPT (*n* = 6515)	Non-PHPT (*n* = 692,642)	Total (*n* = 699,157)
Stroke	women	462 (7.1%) *	13,206 (1.9%)	13,668 (1.9%)
	men	331 (5.1%) *	12,432 (1.8%)	12,763 (1.8%)
	total	793 (12.2%) *	25,638 (3.7%)	26,431 (3.8%)
Ischemic heart disease	women	453 (6.9%) *	12,412 (1.8%)	12,865 (1.8%)
	men	545 (8.4%) *	26,166 (3.8%)	26,711 (3.9%)
	total	998 (15.3%) *	38,578 (5.6%)	39,576 (5.7%)
Atrial fibrillation	women	884 (13.6%)	19,310 (2.8%)	20,194 (2.9%)
	men	612 (9.4%) *	19,867 (2.9%)	20,479 (2.9%)
	total	1496 (23.0%) *	39,177 (5.7%)	40,673 (5.8%)
Deep vein thrombosis	women	588 (9.0%) *	15,528 (2.2%)	16,116 (2.3%)
	men	361 (5.6%) *	13,759 (2.0%)	14,120 (2.0%)
	total	949 (14.6%) *	29,287 (4.2%)	30,236 (4.3%)
Pulmonary embolism	women	356 (5.5%) *	9424 (1.4%)	9780 (1.4%)
	men	190 (3.3%) *	8205 (1.1%)	8395 (1.2%)
	total	546 (8.4%) *	17,629 (2.5%)	18,175 (2.6%)

* *p* < 0.001 (PHPT vs. non-PHPT).

**Table 3:** Uni- and multivariate regression analysis showing the relative risk with 95% confidence interval and statistical significance for various cardiovascular diseases in patients with primary hyperparathyroidism (PHPT).

	Univariate			Multivariate *		
	Odds Ratio	95% CI	*p*	Odds Ratio	95% CI	*p*
Stroke	3.606	3.344–3.888	<0.001	1.494	1.382–1.616	<0.001
Ischemic heart disease	3.067	2.865–3.283	<0.001	1.287	1.195–1.385	<0.001
Atrial fibrillation	4.972	4.689–5.272	<0.001	1.943	1.822–2.073	<0.001
Deep vein thrombosis	3.862	3.601–4.141	<0.001	1.462	1.357–1.575	<0.001
Pulmonary embolism	3.502	3.205–3.828	<0.001	1.576	1.438–1.727	<0.001

* After adjusting for age, gender, hypertension, dyslipidemia, diabetes mellitus, and smoking habit.

## Data Availability

Data available upon reasoned request.
